# Saturated Fatty Acids in Wool as Markers Related to Intramuscular Fat Content in Lambs

**DOI:** 10.3390/ani14192822

**Published:** 2024-09-30

**Authors:** Karla Inostroza, Giovanni Larama, Mario Díaz-Matus de la Parra, Silvana Bravo, Romina Rodríguez, Ana Guerrero, David Cancino-Baier

**Affiliations:** 1 Departamento de Ciencias Agropecuarias y Acuícolas, Facultad de Recursos Naturales, Universidad Católica de Temuco, Temuco 4780000, Chile; 2 Biocontrol Research Laboratory and Scientific and Technological Bioresource Nucleus, Universidad de La Frontera, Temuco 4780000, Chile; giovanni.larama@ufrontera.cl; 3 Escuela de Graduados, Facultad de Ciencias Agrarias y Alimentarias, Universidad Austral de Chile, Valdivia 5090000, Chile; mariodiazmatus@gmail.com; 4 Instituto de Producción Animal, Facultad de Ciencias Agrarias y Alimentarias, Universidad Austral de Chile, Valdivia 5090000, Chile; silvana.bravo@uach.cl; 5 Escuela de Medicina Veterinaria, Facultad de Recursos Naturales y Medicina Veterinaria, Universidad Santo Tomás, Talca 3460000, Chile; rominarodriguezmv@gmail.com; 6 Departamento Producción y Sanidad Animal, Salud Pública Veterinaria y Ciencia y Tecnología de los Alimentos, Universidad Cardenal Herrera-CEU, CEU Universities, Alfara del Patriarca, 46115 Valencia, Spain; ana.guerrerobarrado@uchceu.es; 7 Facultad de Ciencias Agropecuarias y Medioambiente, Universidad de La Frontera, Temuco 4780000, Chile; cancinobaier@gmail.com

**Keywords:** meat quality, behenic acid, lignoceric acid, non-invasive, pasture feeding

## Abstract

**Simple Summary:**

Intramuscular fat (IMF) is related to meat quality and consumer preferences. Several analytical methods involving solvents have been developed to determine IMF and are used to validate rapid and nondestructive new technologies, such as tomography, Raman spectroscopy and NIR. The current study assessed the fatty acid (FA) composition of wool and its relationship to IMF in the *Longissimus lumborum* muscle of lambs. Wool has the potential for non-invasive sampling, reflecting the animal’s metabolism over the past 2 or 3 weeks, and can be stored for long periods of time. The results of this experiment suggest that saturated FA in wool could potentially be used to determine the IMF of lamb.

**Abstract:**

The aim of this study was to identify *Longissimus lumborum* (LL) muscle from lambs with high IMF content (≥3%) using wool FA. The FA composition was characterized in two sections of wool from Suffolk Down lambs, and its relationship with IMF content assessed. One hundred and forty lambs of similar body weight, body condition score, and age were selected. IMF content from LL muscle, and the wool fat and FA composition of the wool were determined. The proportions of FAs in the wool of lambs with low IMF were 51.96 ± 7.3% of saturated, 31.96 ± 7.7% polyunsaturated, and 16.08 ± 2.3% monounsaturated. Similar proportions of FAs were observed in wool samples of lambs with high IMF. Significant differences were found only in the SFA proportion between groups (*p* < 0.05). The predominant FAs in the wool samples were C16:0, C17:0, C18:0, C18:1n9c and C18:2n6c, representing over 60% of total FA methyl esters. Correlations were found between the wool FAs C16:1, C17:0, C18:0, C18:1n9c, C24:0, and C22:6n3 in wool samples divided into two sections. Discriminant analysis identified SFAs, particularly FAs C22:0 and C24:0, as potential candidate for predicting lambs with high IMF content with an accuracy of over 92%. These results provide to our knowledge evidence that IMF content could potentially be determined using wool FAs as a non-invasive method.

## 1. Introduction

Intramuscular fat (IMF) has an important influence on meat palatability associated with juiciness, tenderness and flavour. Consumers desire flavorsome, juicy, tender meat, and currently seek lower fat/healthier food options, two consumer drivers which are linked to the amount of IMF [1]. Also, meat quality is affected by many factors, among which IMF content is recognized as the predominant factor [2]. A certain degree of marbling is essential to achieve consumer satisfaction for palatability, and this parameter increases at the highest rate as fat increases from 1 to 3% [3]. This threshold of 3% of IMF, supported by Lambe et al. [4], was found to deliver acceptable eating quality for lamb loin. Coincident with the above, for a meat cut to be classified as “low fat”, the fat content should be equal to or less than 3% (measured via Soxhlet methodology), however some works on lamb meat have estimated that a threshold over 4% IMF is required to achieve consumer satisfaction for palatability [1,5,6]. Therefore, there is a need to measure the IMF of red meat to ensure its eating quality and sensory attributes, and to meet the expectations of health-conscious consumers.

Due to the importance of IMF in the market and for consumers, it is crucial to develop non-invasive tools for determining IMF in lambs. Several analytical methods have been developed and applied for total lipid extraction from foods, using either single solvents or various solvent mixtures. The Folch-modified method [7] and the official Association of Analytical Chemists (AOAC) method have been widely used for meat and meat products [8]. These methodologies have been used to validate the result of several rapid, non-destructive, and non-invasive technologies that have been investigated, including Raman spectroscopy, computer tomography, Near Infrared (NIR) spectroscopy, etc. [9]. NIR is a cost-effective analytical technique to predict IMF in beef, pork and lamb. However, NIRs presents drawbacks due to its dependence on a reference method, its low sensitivity to minor constituents, limited transfer of calibration between different instruments and complicated spectral data [10]. This is particularly important in some markets, for example, for lamb in Australia, as at present lamb carcass assessments are limited to weight and fat depth over the GR site because lambs are not split or quartered before the fabrication of cuts [11]. Also, these technologies are used to predict IMF content in carcasses, thus a methodology to predict IMF before slaughtering would be advantageous.

In addition to meat, other tissues and fluids have been studied in relation to fat and FA metabolism, i.e., rumen fluid, blood plasma, hair and wool [12,13,14,15]. Wool is a metabolically inert and incremental tissue. It has the advantages that it can be non-invasively collected and conveniently stored at low cost for long periods, and that it reflects an individual’s metabolism retrospectively for 2 or 3 weeks [16,17]. Wool fibers are primarily composed of proteinaceous material (97%), with a low percentage of lipids, polysaccharides, nucleic acids, and mineral salts (3%) [18]. The structure of the wool fiber includes an external, and an internal lipid layer. The external hydrophobic lipid layer, called lanolin or wool wax, is a mixture of esters, diesters, and hydroxy-esters of high molecular weight and is removed during the scouring process. The internal surface of the lipid layer is covalently bonded to the keratin substructure. In the inner hydrophobic fraction–the cortex cells forming the core of the fiber–the lipid components commonly described are free sterols, free fatty acids and polar lipids consisting of cholesterol sulfate and sphingolipids; these are mainly composed of FA with carbon number ranging from 16 to 20 [18,19]. Some studies have reported that FAs accumulate with increasing age, and FA concentration tends to increase with hair segment length. Therefore, different cut-offs have been proposed for evaluating the concentration of FAs in hair, based on proximal and distal sections, to determine specifically biomarkers [20,21,22]. Hair-wool are primarily composed of saturated FAs (SFAs), similar to lamb meat, with a smaller proportion of monounsaturated (MUFA) and polyunsaturated (PUFA) [14,15,23,24,25]. The fat content in wool is similar to hair from cows [26]. Previous studies have established a relationship of the FA profile of hair and wool with the energy availability of early lactating cows [14], and with high milk fat content in ewes [15], respectively. However, the relationship between wool FA and IMF content is not yet known. According to the literature data, we hypothesize that the IMF content in the *Longissimus lumborum* muscle could be reflected in the FA composition of wool. Thus, the aim of this study was to analyze the FA composition of lamb wool in two different segments and to identify *Longissimus lumborum* muscle with high IMF content using wool FAs.

## 2. Materials and Methods

### 2.1. Sample Collection

The study’s method and procedures were approved by University of La Frontera Scientific Ethical Committee (Ethical Review Number 101_17). This study was performed on 140 loin samples. To obtain significant differences in the IMF content (low < 3% and high ≥ 3%) among the groups, a total of 200 male and female lambs aged 4 ± 0.5 months old out of Suffolk Down ewes were identified in three farms in the Araucanía region of Chile (38°54′ S, 72°40′ W). The lambs were slaughtered during the summer. From birth, the lambs were reared on pastures predominantly composed of perennial ryegrass (*Lolium perenne* L.) and white clover (*Trifolium repens* L.). They were transported to a commercial abattoir, held in lairage overnight and slaughtered the following morning at an average weight of 34.33 ± 3.7 kg and body condition score 2.5 ± 0.5 [27], according to standard commercial procedures and welfare codes of practice. At 24 h post-mortem, 100 g samples of *Longissimus lumborum* (LL) muscle were collected from the left side of each carcass. The samples were vacuum-packed, identified in the slaughterhouse, transported to the laboratory, and then frozen at −20 °C until analysis.

For all lambs, one wool sample was taken from the superior left-side hind leg before slaughter. The wool was cut near the skin and stored individually in labeled paper bags at −20 °C until analysis. 

### 2.2. IMF Determination

Prior to analysis, the LL muscle samples were thawed at 4 °C for 12 h and minced individually in liquid nitrogen using a universal grinder. To quantify the IMF content in meat, solvent extraction was used. In this study, the IMF were extracted using a modified Folch method [7]. Briefly, each sample was divided into three homogenous 1 g subsamples, and the fat extraction was performed with 20 mL hexane/isopropanol (2:1 *v*/*v*) (Merck, Darmstadt, Germany). The samples were magnetically shaken and filtered. Fat was retained in the hexane layer. The fat content was determined gravimetrically after complete total solvent evaporation under vacuum in rotavapor (37 °C) and expressed as a percentage. Analyses were performed in triplicate. The meat samples were classified into two groups by IMF content: lambs with a high or low IMF content at a threshold of ≥ or <3%, respectively. This threshold was determined according to values reported by the literature related to meat quality and consumer analysis [1,3,4,5,6].

### 2.3. Wool Analysis

The sequence for performing the analysis of wool FA composition was cleaning, grinding, lipid extraction, methylation, and determination of fatty acid methyl esters (FAME). Before internal-lipid extraction, all wool samples were cleaned and dried [14,26] to remove the surface lipids and contaminants. Each sample of 38 ± 0.3 mm of length was cut in half, resulting in two segments: one near the follicle (proximal section) and the other at the distal end, to measure their FA concentration separately. 1 g of clean wool from near the follicle and 1 g from the distal section were analyzed separately, cut, and ground in a mortar with liquid nitrogen. The wool lipids were extracted using a modified Folch method [7] as described above. FAMEs were prepared according to IUPAC 2.301 [28] protocol. Briefly, 1.3 mL potassium hydroxide in 2 N methanol (Merck, Darmstadt, Germany) and 0.8 mL n-hexane (Merck, Darmstadt, Germany) were added for lipid extraction, followed by shaking for 30 min. The supernatant was filtered with anhydrous sodium sulfate (Merck, Darmstadt, Germany) and transferred into a vial for direct injection, and analyzed by GC using a Clarus 500 model (Perkin Elmer, Buckinghamshire, UK), equipped with a fused silica capillary column SP^TM^ 2380 (60 m × 0.25 mm × 0.2 µm film thickness) (Supelco, Bellefonte, PA, USA), and a flame ionization detector (FID). The thermal program used an initial temperature of 150 °C and maintained for 1 min; the temperature was subsequently increased to 168 °C, at a rate of 1 °C min^−1^ and maintained for 11 min, and finally increased to 230 °C at 6 °C min^−1^ and maintained for 8 min. The injection volume of the sample was 1 µL. The temperature of the injector and FID was 250 °C, and nitrogen was used as the carrier gas, at a flow rate of 1 mL min^−1^. The peak areas were integrated using TotalChrom Workstation software (version 6.3) in post-run analysis. Finally, FAMEs were individually identified by comparing their retention times with those of a commercial standard FAME Mix C4-C24 (Supelco, Pennsylvania, PA, USA), which contains 37 FAMEs, and analyzed under the same chromatographic conditions. The wool FAs were quantified using an internal standard, Nonadecanoic Acid Analytical Standard (C19:0; Merck Darmstadt, Darmstadt, Germany), which was added to each sample and the standard mixture.

### 2.4. Statistical Analysis

Significant differences between low and high IMF content groups and wool sections in FAs, ratios, wool fat, and IMF, were determined using *t*-testing. Pearson’s correlation coefficients were calculated to evaluate the relationship between the wool FA composition in the different sections and IMF in LL muscle. All statistical analyses were performed using SPSS Statistical Software (IBM SPSS Statistical for Windows, version 23.0. Armonk, NY, USA: IBM Corp.), with a significance level of *p* < 0.05. Wool FAs and wool fat content were subjected to discriminant analysis based on IMF content. The classification procedure was performed using a linear discriminant function, using the combination of these variables, and their performance was evaluated by cross-validation. All these functions in addition to the accuracy and Matthews correlations coefficient (MCC), were calculated using the MASS and CARET libraries in the R environment, as detailed in [29,30].

## 3. Results

There were differences in the FA composition of wool from lambs with different IMF content. The groups were classified into high and low IMF categories according to their IMF percentage, which was correlated with the FA composition of the wool samples divided into proximal and distal sections. The IMF contents of the two groups differed significantly (*p* < 0.05) (Table 1). The average fat content of the lamb wool was 2.86%. In wool samples from lambs with low IMF, the overall proportions of FA were as follows: 758.16 ± 10.16 (51.96 ± 7.3%) saturated FA (SFA), 464.64 ± 9.45 (31.96 ± 7.7%) polyunsaturated FA (PUFA), and 235.03 ± 3.43 (16.08 ± 2.3%) monounsaturated FA (MUFA) of the total FAMEs. In wool samples from lambs with high IMF, the FA proportions were similar, with 820.44 ± 17.51 (52.78 ± 8.6%) SFA, 496.48 ± 13.85 (31.60 ± 6.8%) PUFA and 241.69 ± 5.08 (15.65 ± 3.0%) MUFA, on average of total FAMEs. Significant differences between IMF categories were observed only in the SFA proportion (*p* = 0.002).

For each IMF group, several FAs differed significantly between wool sections (*p* < 0.05). The predominant FAs in the wool samples were C16:0, C17:0, C18:0, C18:1n9c and C18:2n6c. In the proximal section of wool samples with low IMF these FAs represent 67.70% of total FAMEs, whereas in the distal section they represented only 60.22%. The FA C18:2n6c did not show differences between wool sections in the low IMF group (*p* > 0.05). In contrast, in the proximal section with high IMF, these FAs represent 61.38% of total FAMEs. In addition, the FAs C13:0, C14:0 and C14:1 contributed significantly to total FAMEs in the proximal section with high IMF (*p* > 0.05), differing from the other sections. In the distal section of wool with high IMF, the five majority FAs described above represent 56.70% of total FAMEs. The five majority FAs were found at higher concentrations in the proximal compared to the distal section (*p* < 0.05) in the low IMF group. Additionally, FAs such as C13:0, C14:0, C15:0, C16:1, C20:0, C22:0 and C22:6n3, which were presented in smaller concentrations, also showed significant differences between wool sections (*p* < 0.05). In samples with high IMF content, the FAs C18:3n3 and C22:6n3 did not show significant differences between wool sections (*p* > 0.05).

The SFA, MUFA and PUFA proportions and desaturase index (DI), differed significantly between wool sections based on IMF content (*p* < 0.05). For each IMF content category, the proportion of SFA and MUFA was significantly higher in the proximal compared to the distal section (*p* < 0.05). The PUFA proportion was higher only in the proximal section with high IMF content (*p* > 0.05), while no significant differences in PUFA proportion were observed between wool sections with low IMF content (*p* > 0.05). The DI C16 values were higher in the distal section for both low and high IMF samples. Moreover, DI C18 values were higher in the proximal section in both IMF groups. However, DI C14 showed a significant difference only in the distal section for samples with low IMF content (*p* < 0.05).

For each wool section, Pearson’s correlations were calculated to determine the relationship between wool FAs and IMF (Table 2). The FAs C10:0, C16:1, C17:0, C22:0 and C22:6n3 were the only FAs that demonstrated a positive correlation between each wool section and IMF content. In contrast, negative correlations were determined for SFAs (C18:0 and C24:0) and MUFA (C18:1n9c) in relation to both wool sections and IMF. In the proximal section of wool, positive correlations were found for SFAs (C12:0, C13:0, C14:0, C15:0 and C20:0), MUFA (C14:1) and PUFAs (C20:3n6 and C18:2n9c). Moreover, in the distal section, only the MUFA (C24:1n9) showed a positive correlation with IMF. The proportions of SFA and PUFA presented a positive correlation in the proximal section but a negative correlation in the distal section. Notably, there was a positive correlation between wool fat in the proximal section and IMF content, which was not observed in the distal section.

The wool FAs from both sections were evaluated to perform discriminant analysis. The SFAs C22:0, C15:0 and C14:0 were the most discriminating variables in the proximal segment of wool samples for classifying high IMF content (Table 3). In the validation, the FA C22:0 demonstrated an accuracy of 95.24% as a single factor. A similar accuracy was achieved using the combination of the FAs C14:0, C15:0 and C22:6n3 for prediction.

The FAs C24:0, C22:6n3 and C17:0 were the most discriminating variables in the distal section for classifying high IMF content (Table 4). FA C24:0 demonstrated the highest accuracy in the validation, achieving 98.00% as a single factor. The best combination of two variables, C17:0 and C22:6n3, revealed an accuracy of 95.24%.

## 4. Discussion

Determining traits of economic and health importance in lamb meat, such as IMF and FA composition, is crucial for assessing eating quality and sensory attributes, as well as for evaluating different grading systems. Consequently, the development of a non-destructive, non-invasive method for measuring the IMF content in lamb meat is of significant interest for the industry, given the impact of IMF on eating quality and health factors [31,32]. Several studies have mentioned the use of NIRs technology for predicting the IMF content in lamb loin [9,10,31,32]; however these have presented variations dependent on the operator or on sample processing, as well as equipment variation, resulting in differences between spectra or absorbance characteristics. Therefore, it is important to develop other ways of predicting IMF content in commercial situations to assess different carcass characteristics. In view of this, indirect parameters such as FAs have been used to determine cows’ health status and quality products in ewes in different production statuses. Metabolites such as FAs have been determined in cow’s hair [14,16,33] and used to assess the cow’s energy status. Furthermore, the FA composition of wool was found to be effective in predicting the milk fat content in ewes’ milk [15]. Wool FAs can originate from four major pathways: directly from the diet, de novo synthesis, formation in the rumen by hydrogenation or bacterial degradation, and release from body fat stores [34]. Therefore, several factors can affect wool FA composition, and probably the energy status plays an important role. As the energy balance decreases and becomes negative, it causes a lower synthesis of short-chain FA and increased mobilization of adipose tissue FA into the blood, particularly long-chain FA [35,36]. However, IMF is considered a late maturing tissue with levels that increase as the animals develop and mature [3,37]; many efforts have been made to improve the IMF content in sheep by nutritional regulation or changing feeding patterns [2].

FAs are incorporated in hair from the external lipid layer and accumulate with increasing age of the hair from the different sources mentioned above. Several studies from forensic science have determined a group of FAs as alcohol biomarkers in hair [20,21,22,38], and the FAs concentration can be evaluated through different sections of hair. The authors concluded that the incorporation of FAs in the hair matrix depends on the distance from the hair root, leading to a typical concentration profile that increases from proximal to distal in human hair [20,38]. In this study, the concentration of wool FAs was evaluated in the proximal and distal sections of lamb wool fibers with low or high IMF content. Contrary to human hair studies, a higher concentration of FAs was found in the proximal than in the distal section. There are several possible explanations for the differences, such as total wool length and damage to wool structure. Additionally, wool fat content was determined for each section. Inostroza et al. [15] reported that the total fat content from lactating ewes varied between 1.12% and 1.25%. Similar findings were reported by Moeller et al. [14] in hair from lactating cows, with values which between 0.40% and 1.64%. In contrast, in the present study, it was observed that the highest values for wool fat content varied between 2.6% and 3.4%. Coincident with other studies [14,15,16], the wool FA composition in lambs is composed mainly of SFA, with smaller proportions of MUFAs and PUFAs.

The main wool FAs identified in wool lipids are C16:0, C18:0, C18:1, C20:0, C22:0, and C24:0 [26]. In the wool samples from lambs with low IMF content, the FAs C16:0 and C18:0 represent together between 56.80 and 46.89% of total wool FAs in the proximal and distal sections, respectively. However, in wool samples from lambs with high IMF content, the FAs C16:0 and C18:0 only represent 40.45–42.83% of total wool FAs in the proximal and distal sections, respectively. This can be explained because other FAs with an odd number of carbon atoms, like C17:0, present a higher concentration than is found with C18:0. Additionally, FAs C12:0–C16:0, which are products of de novo synthesis, are related to energy intake and consequently to the rate of de novo synthesis [24].

The concentration of SFAs and MUFAs were lower in wool from lambs with low IMF content than in the high IMF group (*p* < 0.05). Several studies have characterized the relationship between IMF content and FA composition, finding that IMF showed a high positive correlation with SFA and MUFA, and a high negative correlation with PUFA, n-6, and n-3 FAs [2,3,6]. This may be a result of the contribution of phosphatidylcholine, the main phospholipid in meat and a component of the cell membrane because it acts as a reservoir for PUFA [39]. Duckett et al. [40] in a concentrate diet experiment on nutrient composition in beef cattle, reported that the percentage contribution of phospholipids to total lipids declined greatly with advancing time of feed, decreasing the PUFA content in *longissimus* muscle, while MUFA increased and SFA was relatively unaffected. Zhang et al. [2] showed that when pigs had a higher IMF content, the degree of unsaturation was lower. Individual FA content in lamb meat changes as IMF increases. The increase in C14:0 is associated with a high relative increase in C10:0 and C14:0, which may also be related to elongation of C12:0 FA [6]. These changes in FAs were also determined in the wool of lambs with low or high IMF content, particularly C14:0 and their corresponding MUFA, C14:1; higher concentrations were found in wool samples of lambs with high IMF content. Therefore, the FAs presenting a high correlation with IMF content were mainly SFA and MUFA, which could be reflected in hair FA composition. This is supported because lipid metabolism is thought to play an essential role in the lipid envelope of hair, but is also involved in hair development and function; for example, fatty acid transport protein (FATP) 4 is the primary intestinal FATP and is thought to play a major role in dietary FA uptake [41], but any mutation in FATP4 also leads to defects in hair follicle morphogenesis and hair growth [42].

In our evaluation of FA proportions in wool sample sections from lambs with low or high IMF content, the proximal section has significantly higher content of SFA and MUFA. The study of Suesse et al. [20] analyzed the effect of fatty acid ethyl esters (FAEE) in human hair, to determine more specifically which concentration of FAEE would serve as a biomarkers. Measurements were taken in two different segments (0–3 and 0–6 cm) in order to cover a longer period before sampling; and the FAEE evaluated showed lower values in the 0–3 cm segment (1.54 ± 0.3 ng mg^−1^) than 0–6 cm (1.80 ± 0.4 ng mg^−1^). Contrary to our results, a higher concentration of FA proportions was determined in the proximal than the distal section (*p* < 0.05). The main reasons proposed by the authors are dilution by newly grown hair, and decomposition and elimination of the markers by hair care, among other factors [20]. The difference in the wool section not only affects the FA but also impacts the desaturase index (DI). This index tended to differ between proximal and distal sections (Table 1). The higher DI C14 value in the distal section with low IMF content was likely due to the high IMF content being correlated with a lower degree of unsaturation. A similar pattern was observed with DI C16. Realini et al. [6] observed an increase in the C18 cis-9/C18:0 ratio in lamb meat, suggesting a greater desaturation of C18:0 with increasing IMF%, although the ratios C16:1/C16:0 and C14:1/C14:0 decreased with increasing of IMF%. In our study, however, the highest DI C18 values were observed in the proximal section, regardless of IMF content. Contrary to these results, C18:1n9c concentration increases with IMF content due to enhanced activity of the enzyme delta 9-desaturase, which converts C18:1n9c into C18:0 [24].

A correlation approach was initially tested to determine the relationship between wool FAs and IMF content. The FAs C16:1, C17:0, C22:0, and C22:6n3 maintained a positive correlation in both sections of wool samples, for high or low IMF content. The FA C22:6n3 has also been reported in the hair of lactating cows but it was not significantly correlated with milk performance [14]. Similarly, the FAs C16:1, C17:0, C22:0, and C22:6n3 have been determined in the wool of lactating ewes but were not correlated with milk fat content [15]. Other FAs showed varying correlations, positive or negative, or simply were not correlated with IMF content. However, the correlation analysis of the proximal section indicated that short-chain (C10:0–C15:0) and medium-chain FAs (C16:1–C17:0) had a positive correlation with the IMF content. In contrast, the short-chain FA group exhibited higher variability in the distal section. As mentioned above, these FAs are important as part of de novo FA synthesis, and subsequently can only be produced and stored in the hair or wool if sufficient energy is available for further de novo synthesis [14,16]. Furthermore, analysis of the proximal section of wool samples may reflect the energy status of the lambs. The long-chain FAs (LCFA) C18:0 and C18:1n9c showed a negative correlation with IMF content. Other studies have reported that the FA C18:1n9c in wool exhibited a strong positive correlation with milk fat content in ewes at 30 and 60 days in milk [15], or did not find correlations between energy intake and the production of FAs C18:1 or C18:0 in the hair of lactating cows [33]. Positive correlations in other LCFA (C18:2n6c, C22:0, and C22:6n3) were determined. Special attention is given to the essential FA C18:2n6c, the most abundant PUFA in pasture-fed lambs, and its relationship with energy intake [24]. Increases in energy lead to higher amounts of C18:2n6c leaving the rumen unfermented; these escape the biohydrogenation processes, and are available for storage, including in hair and wool [33]. In our study, the LCFA C22:0 and C24:0 showed a positive and negative correlation, respectively, in both sections. Inostroza et al. [15] found that the FA C22:0 identified in wool was not correlated with the fat content of ewes’ milk but FA C24:0 was positively correlated with milk fat after 30 days in milk. However, a study examining the variation in FA profiles between different IMF content groups in rams observed that the content of FA C22:0 was lower in the high IMF group compared to the low IMF group, while the contents of other FAs were higher in the high IMF group [2]. The results of the present study suggest that the proximal section provided better information about lambs, as explained by the FA correlations in the different sections with the varying the IMF content.

Discriminant analysis was performed to classify subjects according to high IMF content. The analysis was applied to develop models for the identifying high IMF content lambs based on either a single FA or combinations of FAs in each wool section. In both cases, SFAs emerged as positive candidate markers for IMF content, and, as mentioned above, these FAs are predominant in wool lipid concentration. In the proximal section, the FA C22:0, as a single factor used to classify lambs with high IMF content, demonstrated an accuracy of 95.24%. This accuracy was also achieved with the combination of the FAs C14:0, C15:0, and C22:6n3. Additionally, the maximum accuracy in the distal section was achieved with FA C24:0, reaching 98.00%. FAs such as C22:0 and C24:0, like most SFAs can be synthesized by the body; except n-3 and n-6 considered essential for the function of the body [25]. These FAs are little described in studies related to hair and wool [14,15,16,33], and their comparative accumulation in the proximal and distal sections has not been reported. This is one of the first study to provide evidence about the relationship between wool FA and IMF content, detecting positive markers for this variable. However, several factors can affect the hair-wool FA composition, and the energy status of the animals probably has a crucial role. The negative energy balance (NEB) could increase the mobilization of body fat, as mentioned above, result in body weight loss in animals, and the FA composition could be changed by NEB [43]. Nutrition also influence the FA composition, for example, the fat supplementation are related to the chain length and degree of saturation of FA [44]. Moeller et al. [14] reported that the stress can also affect the FA composition of blood and hair or wool and can inhibit complete de novo FA synthesis. Thus, these factors must be considered when utilizing wool FAs as markers. Finally, FAs with the highest accuracy can be detected in the routine analysis of IMF FA composition for meat quality, conveniently using the same gas chromatography equipment.

## 5. Conclusions

The present study is one of the first to investigate the relationship between FAs composition in different sections of wool samples with IMF content in lambs. The results demonstrate that wool SFAs C22:0 and C24:0 were significantly correlated with IMF content in the different sections of the wool. Discriminant analysis models determined that lambs with high IMF content could be identified based on the concentration of these FAs, depending on the wool section sampled, with an accuracy over 92%, under the conditions in which the study was carried out. Additionally, it is necessary further investigation to evaluate other nutritional conditions of the lambs to validate these SFAs wool markers. The advantage of using wool or hair is its simplicity sampling, applicable on any production farms, and the FA analysis can be associated with meat quality. This study provides valuable information obtained from the FA composition of wool and could be applied to milk and meat products as an alternative non-invasive predictive method.

## Figures and Tables

**Table 1:** Fatty acids (FA) composition (µg/g of wool) of different sections of wool from Suffolk Down lambs.

Fatty Acids	Low IMF ^1^ (*n* = 70)		*p*-Value ^3^	High IMF ^2^ (*n* = 70)		*p*-Value ^3^
	Proximal	Distal		Proximal	Distal	
C10:0	42.93 ± 2.14	42.25 ± 1.90	0.811	52.52 ± 2.52	32.15 ± 1.68	0.001
C12:0	20.51 ± 1.16	22.95 ± 1.13	0.135	32.05 ± 1.46	21.89 ± 1.13	0.004
C13:0	72.12 ± 2.87	85.20 ± 3.09	0.002	112.89 ± 4.94	64.94 ± 3.14	0.034
C14:0	63.17 ± 2.09	66.67 ± 2.17	0.248	116.67 ± 5.59	76.03 ± 4.50	0.000
C14:1	71.78 ± 2.71	91.49 ± 2.78	0.000	113.41 ± 4.51	78.68 ± 4.43	0.005
C15:0	11.00 ± 0.79	21.13 ± 1.07	0.011	40.95 ± 1.92	17.67 ± 0.92	0.022
C16:0	299.71 ± 11.75	224.99 ± 8.36	0.002	265.41 ± 10.60	207.43 ± 8.61	0.012
C16:1	12.85 ± 0.49	17.93 ± 0.69	0.010	20.31 ± 1.01	25.47 ± 1.26	0.002
C17:0	108.39 ± 2.81	89.46 ± 2.32	0.004	179.73 ± 8.55	146.10 ± 4.53	0.001
C18:0	159.73 ± 5.02	106.75 ± 3.03	0.000	129.23 ± 3.89	77.59 ± 2.21	0.004
C18:1n9c	142.25 ± 3.24	69.76 ± 2.52	0.020	113.99 ± 2.81	52.30 ± 1.70	0.003
C18:2n6c	322.61 ± 12.47	346.33 ± 14.32	0.214	459.73 ± 15.63	223.97 ± 8.09	0.020
C20:0	7.40 ± 0.29	11.56 ± 0.53	0.000	16.77 ± 1.07	7.65 ± 0.30	0.000
C18:3n3	35.38 ± 1.11	34.65 ± 1.03	0.633	32.94 ± 1.56	33.22 ± 1.46	0.899
C22:0	4.71 ± 0.17	5.82 ± 0.22	0.002	14.50 ± 0.73	6.98 ± 0.28	0.002
C20:3n6	40.53 ± 1.59	37.61 ± 1.27	0.155	54.19 ± 2.73	37.99 ± 1.65	0.001
C22:2	35.75 ± 1.17	34.90 ± 1.21	0.616	25.51 ± 1.36	34.54 ± 1.12	0.010
C24:0	19.19 ± 0.76	30.67 ± 0.93	0.000	14.67 ± 0.65	7.07 ± 0.27	0.000
C24:1n9	31.39 ± 1.39	32.62 ± 0.92	0.463	32.08 ± 1.21	47.14 ± 1.88	0.003
C22:6n3	23.98 ± 0.98	17.56 ± 0.46	0.024	42.85 ± 1.93	48.04 ± 2.24	0.081
FA Proportions						
SFA ^4^	808.85 ± 15.14	707.49 ± 10.61	0.004	975.39 ±19.37	665.51 ± 12.83	0.001
MUFA ^5^	258.25 ± 4.16	211.81 ± 3.81	0.000	279.80 ± 5.98	203.59 ± 5.14	0.000
PUFA ^6^	458.24 ± 12.64	471.05 ± 14.11	0.500	615.22 ± 16.68	377.76 ± 9.30	0.010
Desaturase index						
DI C14 ^7^	0.52 ± 0.01	0.58 ± 0.01	0.002	0.49 ± 0.01	0.51 ± 0.02	0.519
DI C16 ^8^	0.05 ± 0.003	0.08 ± 0.003	0.004	0.07 ± 0.004	0.11 ± 0.001	0.001
DI C18 ^9^	0.48 ± 0.01	0.39 ± 0.001	0.000	0.47 ± 0.01	0.40 ± 0.01	0.000
Fat content Wool fat (g/g of wool)	0.034 ± 0.01	0.027 ± 0.01	0.014	0.027 ± 0.01	0.026 ± 0.01	0.363

^1^ High IMF: high intramuscular fat in LL muscle ≥ 3%. ^2^ Low IMF: low intramuscular fat in LL muscle < 3%. ^3^ *p*-value significant at *p* < 0.05 for *t*-testing analysis. ^4^ SFA: saturated fatty acids, sum of C10:0 + C12:0 + C13:0 + C14:0 + C15:0 + C16:0 + C17:0 + C18:0 + C20:0 + C22:0 + C24:0. ^5^ MUFA: monounsaturated fatty acids, sum of C14:1 + C16:1 + C18:1n9c + C24:1n9. ^6^ PUFA: polyunsaturated fatty acids, sum of C18:2n6c + C18:3n3 + C20:3n3 + C22:2 + C22:6n3. ^7^ DI C14: desaturase index C14 = C14:1/(C14:0 + C14:1). ^8^ DI C16: desaturase index C16: desaturase index C16 = C16:1/(C16:0 + C16:1). ^9^ DI C18: desaturase index C18 = C18:1n9c/(C18:0 + C18:1n9c).

**Table 2:** Pearson’s correlation coefficients among wool fatty acids content and intramuscular fat in muscle of Suffolk Down lambs.

Fatty Acids	Intramuscular Fat Content	
	Proximal (*n* = 70)	Distal (*n* = 70)
C10:0	0.224 **	−0.234 **
C12:0	0.298 **	−0.015
C13:0	0.403 **	−0.238 **
C14:0	0.503 **	0.148
C14:1	0.306 **	−0.144
C15:0	0.582 **	−0.131
C16:0	−0.108	−0.124
C16:1	0.396 **	0.224 **
C17:0	0.379 **	0.514 **
C18:0	−0.322 **	−0.452 **
C18:1n9c	−0.376 **	−0.309 **
C18:2n6c	0.302 **	−0.485 **
C20:0	0.423 **	−0.348 **
C18:3n3	−0.031	−0.132
C22:0	0.521 **	0.175 *
C20:3n6	0.336 **	0.007
C22:2	−0.396 **	−0.137
C24:0	−0.319 **	−0.700 **
C24:1n9	−0.073	0.404 **
C22:6n3	0.361 **	0.503 **
SFA ^1^	0.380 **	−0.107 *
MUFA ^2^	0.063	−0.075
PUFA ^3^	0.332 **	−0.436 **
Wool fat	0.286 **	−0.040

^1^ SFA: saturated fatty acids. ^2^ MUFA: monounsaturated fatty acids. ^3^ PUFA: polyunsaturated fatty acids. Correlation coefficients were significant at level: * *p* < 0.05, ** *p* < 0.01.

**Table 3:** Discriminant analysis of wool FAs and intramuscular fat content in the proximal section of wool samples.

Variable	True	False	False	True	MCC ^1^	Accuracy
	Positive	Positive	Negative	Negative		
C22:0	18	0	3	21	0.857	92.86%
C14:0	15	2	6	19	0.619	85.95%
C15:0	17	4	2	19	0.714	80.71%
C22:6n3	19	2	2	19	0.333	66.67%
C14:0 + C15:0 + C22:6n3	19	0	2	21	0.904	95.24%

^1^ Matthews correlations coefficient.

**Table 4:** Discriminant analysis of wool FAs and intramuscular fat content in the distal section of wool samples.

Variable	True	False	False	True	MCC ^1^	Accuracy
	Positive	Positive	Negative	Negative		
C24:0	20	0	1	21	0.952	98.00%
C22:6n3	17	0	6	19	0.714	85.71%
C17:0	18	3	4	17	0.666	83.33%
C18:0	19	0	3	20	0.381	69.05%
C17:0 + C22:6n3	19	0	2	21	0.904	95.24%

^1^ Matthews correlations coefficient.

## Data Availability

All data obtained in this study are available in the article.
